# Current knowledge on the *Ralstonia solanacearum* type III secretion system

**DOI:** 10.1111/1751-7915.12056

**Published:** 2013-04-26

**Authors:** Núria S Coll, Marc Valls

**Affiliations:** 1 Centre for Research in Agricultural Genomics (CRAG).Edifici CRAGCampus UAB, 08193, Bellaterra, Catalonia, Spain; 2 Department of Genetics, Universitat de BarcelonaAv. Diagonal 643 Annex, 08028, Barcelona, Catalonia, Spain

*Ralstonia solanacearum* was ranked in a recent survey the second most important bacterial plant pathogen, following the widely used research model *Pseudomonas syringae* (Mansfield *et al*., 2012). The main reason is that bacterial wilt caused by *R. solanacearum* is the world's most devastating bacterial plant disease (http://faostat.fao.org), threatening food safety in tropical and subtropical agriculture, especially in China, Bangladesh, Bolivia and Uganda (Martin and French, 1985). This is due to the unusually wide host range of the bacterium, its high persistence and because resistant crop varieties are unavailable. In addition, *R. solanacearum* has been established as a model bacterium for plant pathology thanks to pioneering molecular and genomic studies (Boucher *et al*., 1985; Salanoubat *et al*., 2002; Cunnac *et al*., 2004b; Occhialini *et al*., 2005; Mukaihara *et al*., 2010). As for many bacterial pathogens, the main virulence determinant in *R. solanacearum* is the type III secretion system (T3SS) (Boucher *et al*., 1985; 1994), which injects a number of effector proteins into plant cells causing disease in hosts or a hypersensitive response in resistant plants. In this article we discuss the current state in the study of the *R. solanacearum* T3SS, stressing the latest findings and future perspectives.

## A regulatory cascade controls T3SS expression

Synthesis of the T3SS machinery – encoded by some 20 *hrp/hrc* genes – is tightly controlled in all species studied, probably due to its high metabolic cost. *Ralstonia solanacearum* is the only bacterial species for which a regulatory cascade linking T3SS gene expression to plant host contact has been described (Brito *et al*., 2002). In *R. solanacearum hrp/hrc* gene induction is triggered upon recognition of an unidentified non-diffusible cell wall component by the outer membrane receptor PrhA (Aldon *et al*., 2000), which transfers the activation signal through a cascade of transcriptional regulators (Brito *et al*., 2002). HrpG is a central regulator in this cascade (Brito *et al*., 1999; Valls *et al*., 2006), whose downstream activator HrpB directly controls transcription of the T3SS genes and its associated effectors (Genin *et al*., 1992; Occhialini *et al*., 2005). Interestingly, these two regulators have homologues in various *Xanthomonas ssp.* and *Burkholderia ssp.* strains, including the human pathogen *B. pseudomallei* (Wengelnik and Bonas, 1996; Zou *et al*., 2006; Li *et al*., 2011; Lipscomb and Schell, 2011), whereas the PrhA receptor and the upper regulators in the cascade are not conserved in other species.

## A regulatory network with connections to many cellular processes

In addition to the activation by the presence of plant cell wall components, expression of the T3SS genes is also induced by metabolic and environmental inputs. It has been known for a long time that *hrpB* expression is repressed when the bacterium grows in complete medium, as compared with a minimal medium that is thought to mimic plant apoplastic fluids (Arlat *et al*., 1992; Genin *et al*., 2005). More recently, it was found that other regulatory circuits impact *hrp* gene expression. The global regulator PhcA, which activates expression of many virulence activities including motility, plant cell wall degradation, and exopolysaccharide synthesis (Genin and Denny, 2012) has been reported to repress *hrpB* expression by two orders of magnitude during growth in complete medium (Genin *et al*., 2005). PhcA can also bind directly to the promoter of upstream regulators in the *Hrp*cascade but it only downregulates their transcription to one half of the normal levels (Yoshimochi *et al*., 2008). Recent findings showed that PrhG – a HrpG paralogue – also influences expression of the HrpB regulon (Plener *et al*., 2010) and that this pathway is modulated by an unrelated virulence operon (Zhang *et al*., 2011). Thus, the actual view is that of a complex network of regulators controlling *hrp* gene expression in connection with a number of environmental and physiological cues.

The *hrp* regulatory system thus integrates different inputs but it also brings about various output responses by co-regulating transcription of the T3SS and effector genes to that of genes likely associated to metabolic adaptation to parasitic life in the plant (Occhialini *et al*., 2005; Valls *et al*., 2006). Indeed, transcriptomic studies have revealed that HrpG controls expression of some 400 genes, half of them independently of the downstream regulator HrpB. Some of these additional genes encode lectins and enzymes that degrade plant polysaccharides or drive the synthesis of polyamines or phytohormones (Valls *et al*., 2006). Further analyses may detect additional targets of the T3SS regulatory system that have escaped our notice due to experimental or technical limitations. In this sense, it is expected that RNA sequencing experiments can identify small RNAs involved in virulence controlled by the *hrp* regulators, as has been found in *Xanthomonas campestris*, which bears a closely-related regulatory system (Chen *et al*., 2011; Schmidtke *et al*., 2012).

## T3SS regulation *in planta*

An experimental limitation of the above described regulatory circuits is that they were all defined based on experiments carried out *in vitro* using synthetic media. Recent research has focused on determining their relevance and expression timing *in planta* during infection. The creation of a gene delivery system to integrate gene constructs in a permissive site of the *R. solanacearum* chromosome (Monteiro *et al*., 2012b) has been key to monitor transcription in these conditions. This tool enables the analysis of promoter output from single-copy fusions to fluorescent or luminescent reporters during plant infection, as the constructs remain stably integrated in the modified strains. Surprisingly, the master T3SS regulator *hrpB* was found to be transcribed in bacteria growing inside wilting plants, causing expression of *hrp* genes under these conditions (Monteiro *et al*., 2009; 2012a). These findings have been recently validated by an independent transcriptome analysis approach, which has confirmed that half of the HrpB regulon is induced in bacteria recovered from wilting plants (Jacobs *et al*., 2012). These results are in contradiction with the widespread view that the T3SS is only required during the first stages of host colonization. This notion was based on the observations that the T3SS genes are induced immediately after plant contact (Kamoun and Kado, 1990; Thwaites *et al*., 2004; Ortiz-Martin *et al*., 2010) and that this system is involved in suppression of host defence responses to promote bacterial multiplication early after infection (Deslandes and Rivas, 2012). Thus, it will be interesting to ascertain whether the T3SS remains active in late stages of disease development in other plant pathogens or if this is a particularity of *R. solanacearum*, and to elucidate what is the functional importance of the T3SS – if any – during the *R. solanacearum* saprophytic life cycle.

## A large effector repertoire

One of the key questions in bacterial pathogenicity is defining the whole inventory of the type III effectors (T3E) present in a given strain or species. The pioneering genome sequencing and annotation of *R. solanacearum* strain GMI1000 identified a first set of effector candidate genes based on homology to known effectors from other species or presence of domains typically eukaryotic (Salanoubat *et al*., 2002). The existence of well-defined T3SS transcriptional regulators greatly contributed to complete the list. Two approaches were followed to identify candidate effectors co-regulated with the T3SS: (i) the search for promoters with a HrpB binding sequence, similar to the PIP box described in *X. campestris* (Cunnac *et al*., 2004a; Koebnik *et al*., 2006) and (ii) transcriptomic studies using HrpB-deficient and overexpressing strains (Occhialini *et al*., 2005). Translocation analyses with the *cyaA* reporter or T3SS-dependent secretion to the medium have been used to validate most effector candidates (Cunnac *et al*., 2004b; Tamura *et al*., 2005; Mukaihara *et al*., 2010; Solé *et al*., 2012), so that the reference strain GMI1000 is thought to bear 72 type III effectors (Poueymiro and Genin, 2009; Mukaihara *et al*., 2010). Compared with animal pathogens, bacterial plant pathogens contain larger numbers (∼ 30–40) of effectors, but the *R. solanacearum* effector repertoire is exceptionally large, probably due to its wide host range.

A pan-genomic analysis of *R. solanacearum* will determine the super-effector repertoire and help define core and variable effectors in this species, providing evolutionary cues on host range determination. A recent study comprising 19 *P. syringae* strains yielded a super-repertoire of 57 effector genes (Baltrus *et al*., 2011). Considering that the average effector number per strain analysed is considerably lower in *P. syringae* compared with *R. solanacearum* (15–30 in *P. syringae* versus 72 in *R. solanacearum* GMI1000), it is reasonable to expect that the super-effector repertoire of *R. solanacearum* will be correspondingly larger. Up to date, the genomes of 11 *R. solanacearum* strains have been sequenced (GMI1000, RS1000, UW551, Po82, CFBP2957, PSI07, CMR15, Molk2, IPO1609, K60 and Y45) and many others are on their way. These genomes, representative of the whole range of strains composing the *R. solanacearum* species complex, will facilitate pan-genomic analyses in the near future and shed light on effector conservation and function in this species. It will be interesting to ascertain whether in *R. solanacearum* divergent repertoires can be found in strains that are pathogenic on the same host, as it is the case for *P. syringae* (Baltrus *et al*., 2011; Lindeberg *et al*., 2012).

The minimal functional set of core effectors has not been yet determined in *R. solanacearum*. In *P. syringae* DC3000 it has been recently shown to comprise AvrPtoB, HopE1, HopG1, HopAM1, AvrE, HopM1, HopAA1 and HopN1 (Cunnac *et al*., 2011). These effectors function together in host immune suppression, chlorosis and lesion formation, in addition to bacterial growth. Among these, HopG1 is the most widespread in *R. solanacearum* sequenced strains, being only absent in PSI07, K60 and Y45 HopAA1 is the second most represented, as it can be found in GMI1000, Po82, Molk2, IPO1602, CFBP2957 and CMR15. AvrE homologues are identified in Po82, Molk2, IPO1602 and CFBP2957, although the picture is more complex, as distantly-related orthologues may be present in other strains. Finally, an AvrPtoB homologue is only present in Molk2 and the remaining four *P. syringae* predicted core effectors (HopM1, HopN1, HopE1, HopAM1) are absent in *R. solanacearum*. The fact that only half of the *P. syringae* core effectors have members in *R. solanacearum* may indicate that the core effectome in this species is constituted by either functional analogues with no sequence similarity to their *P. syringae* counterparts or by a total different set of activities. Functional genetics studies will clarify in the future which of these hypotheses is true.

## Type III effector function

Deciphering effector function is essential to understand the molecular interactions between pathogens and their hosts in terms of host specificity and pathogenicity. In *P. syringae*, it has been suggested that a small subset of core effectors target antimicrobial vesicle trafficking in plants, whereas a larger and more variable set would interfere with plant kinase-based pathogen recognition pathways (Lindeberg *et al*., 2012). Whether these two strategies to defeat plant immune processes are conserved in *R. solanacearum* remains an open question.

Up to date 23 *R. solanacearum* T3E have been assigned a function *in planta* using biochemical and/or pathology assays (Table 1). To study the contribution of each individual effector to bacterial fitness *in planta*, three methods have been used: (i) to measure growth of *R. solanacearum* mutant strains inside of natural hosts (tomato, eggplant); (ii) to measure growth of *P. syringae* heterologously expressing *R. solanacearum* T3E in Arabidopsis (Solé *et al*., 2012); (iii) competitive index assays between co-inoculated wt and mutant strains, which have proved to be a highly sensitive method to detect minor contributions to pathogenicity (Macho *et al*., 2010). These methods have revealed that several effectors promote growth in *R. solanacearum* natural hosts (Table 1): AvrPphF, AWR1, AWR2, PopP2 and Rip34 (HopD1-like) in tomato; AvrPphF, AWR1, AWR2, Rsp0842 (PopC-like), PopP2, SKWP4, Rip19 (AvrBs3-like), Rip39, Rip64, Rip3, Rip55 and Rip23 in eggplant; and AvrPphF, PopP2, Rsp0842 (PopC-like) and Rip34 (HopD1-like) in bean. Interestingly, two members of the AWR family show contrasting phenotypes, restricting growth in Arabidopsis and tomato (AWR4) or eggplant and Arabidopsis (AWR5), which may indicate a certain degree of recognition of these T3S in certain host cellular contexts. Other *R. solanacearum* T3E have been ascribed an avirulence function: AvrA is considered the major determinant leading to resistance of tobacco to some strains (Carney and Denny, 1990; Robertson *et al*., 2004; Poueymiro *et al*., 2009). Other avirulence reactions are triggered by PopP1 in resistant tobacco plants and in petunia (Lavie *et al*., 2002), PopP2 in Arabidopsis (Deslandes *et al*., 2002; 2003; Bernoux *et al*., 2008) and AWR2 and AWR5 in various contexts (Solé *et al*., 2012). Together, these results evidence that the interaction of *R. solanacearum* with its different plant hosts partly results from a combination of synergistic and antagonistic interactions between specific effectors within a single strain.

**Table 1:** List of *R. solanacearum* type III effector with a defined role *in planta* or for which the mode of action has been (partly) elucidated

Gene name									
							
GMI1000	RS1000	Alternative name	Protein name	Family	Predicted domains	Role *in planta*	Hosts tested	Mode of action	References
RSc0608	rip5	avrA	AvrA	–	–	Avirulence/Promotes growth	Nicotiana spp./Tomato	–	Carney and Denny (1990); Robertson *et al*. (2004); Turner *et al*. (2009); Macho *et al*. (2009)
RSp0822	rip40	–	AvrPphF	HopF2/AvrPphF	–	Promotes growth	Tomato, Eggplant, Bean	–	Macho *et al*. (2010)
Rsc2139	–	–	Awr1	AWR	–	Promotes growth	Tomato, Eggplant	–	Solé *et al*. (2012)
RSp0099	rip29	hpx31/ripA	Awr2		–	Avirulence/Promotes growth	Nicotiana spp./Tomato, Eggplant, Arabiodpsis	–	
RSp0847	rip45	hpx4	Awr4		–	Restricts growth	Arabidopsis	–	
RSp1024	rip56	hpx10	Awr5		–	Avirulence/Restricts growth	Nicotiana spp./Tomato, Eggplant, Arabiodpsis	–	
RSp0914	rip53	gala1	Gala1	GALA	LRR repeats – F-box	–	–	Interaction with SKP1-like proteins	Angot *et al*. (2006)
RSp0028	rip28	gala3	Gala3			–	–		
RSc1801	rip18	hpx16	Gala5			–	–		
RSc1356	rip13	hpx13	Gala6			–	–		
RSc1357	rip14	hpx14	Gala7			Host specificity factor	Medicago truncatula		
RSp0877	rip49	popA	PopA	–	Harpin		Nicotiana	Formation of plasma membrane ion-conducting pores	Racapé *et al*. (2005)
Rsp0842	–	–	–	PopC	LRR	Promotes growth	Eggplant, Bean	–	Macho *et al*. (2010)
RSc0826	rip7	popP1	PopP1	YopJ/AvrRxv	Ser/Thr acetyltransferase, functional NLS	Avirulence	Petunia	–	Lavie *et al*. (2002)
RSc0868	rip8	popP2	PopP2			Avirulence/Promotes growth	Arabidopsis/Tomato, Eggplant, Bean	Nuclear relocalization of RRS1-R and RD19, binds RRS1-R	Deslandes *et al*. (2002); Deslandes *et al*. (2003); Bernoux *et al*. (2008); Macho *et al*. (2010)
RSc1839	rip20	hpx30	Skwp4	SKWP	Heat/armadillo-related repeats	Promotes growth	Eggplant	–	Macho *et al*. (2010)
RSc1815	rip19	hpx17	Rip19	AvrBs3	central repeat	Promotes growth	Eggplant	–	
RSp0304	rip34	hpx25	Rip34	HopD1/AvrPphD	–	Promotes growth	Tomato, Eggplant, Bean	–	
RSp0732	rip39	hpx27	Rip39	HopAV1	Coiled-coil	Promotes growth	Eggplant	–	
RSp1281	rip64	hpx24	Rip64	HopR1	–	Promotes growth	Eggplant	–	
RSc0257	rip3	–	Rip3	–	Ankyrin repeat	Promotes growth	Eggplant	–	
RSp1022	rip55	hpx21	Rip55	–	–	Promotes growth	Eggplant	–	
RSc2359	rip23	hpx28	Rip23	–	–	Promotes growth	Eggplant	–	

The characterization of the molecular/biochemical function of the increasingly large number of *R. solanacearum* effectors remains a major challenge. So far only a limited number of its T3E have been biochemically characterized (Table 1). Several members of the GALA family (Gala1, Gala3, Gala5, Gala6 and Gala7) have been shown to interact with SKP1-like proteins, and are thought to mimic plant E3 ubiquitin ligases (Angot *et al*., 2006). PopP2 has been shown to trigger re-localization of the cysteine protease RD19 to the nucleus, where it is thought to form a protein complex with the atypical WRKY-containing NB-LRR protein RRS1-R leading to disease resistance (Deslandes *et al*., 2002; 2003; Bernoux *et al*., 2008). However, direct interaction has only been shown for PopP2/RRS1-R and RRS1-R/RD19, but not for PopP2 and RD19. Recent work suggests that RRS1-R activation of the plant immune responses upon PopP2 recognition involves perception of PopP2 auto-acetylation (Tasset *et al*., 2010). Finally, the harpin-like T3E PopA has been shown to localize to the membrane of tobacco cells, where it forms ion-conducting pores, likely facilitating translocation of bacterial proteins into the cytoplasm of plant cells (Racapé *et al*., 2005).

Despite all our current knowledge on *R. solanacearum* T3E derived from the combination of genomic, biochemical and pathology data obtained in the last two decades, there is still a considerable number of effectors with no assigned function. These are usually effectors with no similarity to known proteins or domains or no apparent role in virulence or avirulence. The lack of assigned function *in planta* for many effectors is likely due to redundancy and specialized functionality restricted to certain host plant contexts. To dissect such complex interface between a pathogen and its host a novel genetic screening (insertional mutagenesis and depletion, iMAD) has been successfully used (O'Connor *et al*., 2012). This method systematically combines bacterial and plant mutations, and would be extremely helpful to characterize the interaction of *R. solanacearum* with its multiple hosts. Still, 30% of *R. solanacearum* T3Es have no counterpart in other bacteria (Mukaihara *et al*., 2010), making this species a good model to explore novel effector functions.
